# Evaluating the COVID-19 vaccination program in Japan, 2021 using the counterfactual reproduction number

**DOI:** 10.1038/s41598-023-44942-6

**Published:** 2023-10-18

**Authors:** Taishi Kayano, Yura Ko, Kanako Otani, Tetsuro Kobayashi, Motoi Suzuki, Hiroshi Nishiura

**Affiliations:** 1 Kyoto University School of Public Health, Yoshida-Konoe-cho, Sakyo-ku, Kyoto, 606-8501 Japan; 2 Center for Surveillance, Immunization, and Epidemiologic Research, National Institute of Infectious Diseases, Tokyo, 162-8640 Japan; 3 Department of Virology, Tohoku University Graduate School of Medicine, Miyagi, 980-8575 Japan

**Keywords:** Computational biology and bioinformatics, Infectious diseases, Epidemiology

## Abstract

Japan implemented its nationwide vaccination program against COVID-19 in 2021, immunizing more than one million people (approximately 1%) a day. However, the direct and indirect impacts of the program at the population level have yet to be fully evaluated. To assess the vaccine effectiveness during the Delta variant (B.1.617.2) epidemic in 2021, we used a renewal process model. A transmission model was fitted to the confirmed cases from 17 February to 30 November 2021. In the absence of vaccination, the cumulative numbers of infections and deaths during the study period were estimated to be 63.3 million (95% confidence interval [CI] 63.2–63.6) and 364,000 (95% CI 363–366), respectively; the actual numbers of infections and deaths were 4.7 million and 10,000, respectively. Were the vaccination implemented 14 days earlier, there could have been 54% and 48% fewer cases and deaths, respectively, than the actual numbers. We demonstrated the very high effectiveness of COVID-19 vaccination in Japan during 2021, which reduced mortality by more than 97% compared with the counterfactual scenario. The timing of expanding vaccination and vaccine recipients could be key to mitigating the disease burden of COVID-19. Rapid and proper decision making based on firm epidemiological input is vital.

## Introduction

Vaccination against coronavirus disease (COVID-19) was widely implemented at nationwide and global scale; therefore, its evaluation at population level, including direct and indirect effects, is key for assessing this policy program^1–3^. For instance, Japan implemented a nationwide vaccination program against COVID-19 in 2021 using mRNA vaccines and prioritizing health care professionals from February 2021, then older adults aged ≥ 65 years and those with underlying comorbidities, followed by younger individuals. For mass vaccination, the Pfizer/BioNTech mRNA vaccine (BNT162b2) using ancestral severe acute respiratory syndrome coronavirus 2 (SARS-CoV-2) strain was utilized. The Moderna vaccine (mRNA-1273) was also used for a part of the vaccination rollout and also for vaccination in the workplace. Immunization was conducted to cover more than one million people (approximately 1%) a day when the pace of vaccination was at its peak. Therefore, post-hoc evaluation is essential to understand how influential the program was at population level. Alongside the vaccination program, various public health and social measures (PHSM) were implemented, including the declaration of the state of emergency and contact tracing^4^. These measures aimed to suppress virus transmission even temporarily, thereby alleviating the burden on healthcare facilities and protecting the health infrastructure. Despite these efforts, the virus posed significant challenges, partly due to the emergence of new variants with elevated transmissibility including Alpha (B.1.1.7) and Delta (B.1.617.2) variants, imposing additional difficulties in controlling the spread of SARS-CoV-2^5–7^.

In evaluating the indirect effects of vaccination owing to reduced opportunities for infection and decreased transmissibility (e.g., herd immunity effect), the epidemiological evaluation of population-level effectiveness calls for statistical methods^8–11^. For direct effects only (i.e., whether vaccinated individuals are protected biologically by comparing vaccinated and unvaccinated people), the estimation is simpler, as reported in many countries^12–16^, including estimates in Japan^17^. However, evaluation of population-level effects are scarce (mainly in the United States and Israel)^18,19^, although global estimates have been reported^20^. Whereas the indirect effectiveness of vaccination has been understudied, the related published studies imply that the impact of herd immunity has been substantial during the pre-Omicron period of the COVID-19 pandemic^18,19^.

The present study is focused on the post-hoc evaluation of the vaccination program in Japan where the mortality impact of COVID-19 has been one of the lowest among countries belonging to the Organization for Economic Cooperation and Development^21^. Calculating the counterfactual scenario, herein, we aimed to estimate the total effectiveness of COVID-19 vaccination in Japan in 2021, during which the course of the primary series of the vaccination program was completed and third dose (or booster dose) was not administered yet. We further examined scenarios involving different timing and recipients of vaccination.

## Results

**Figure 1:**
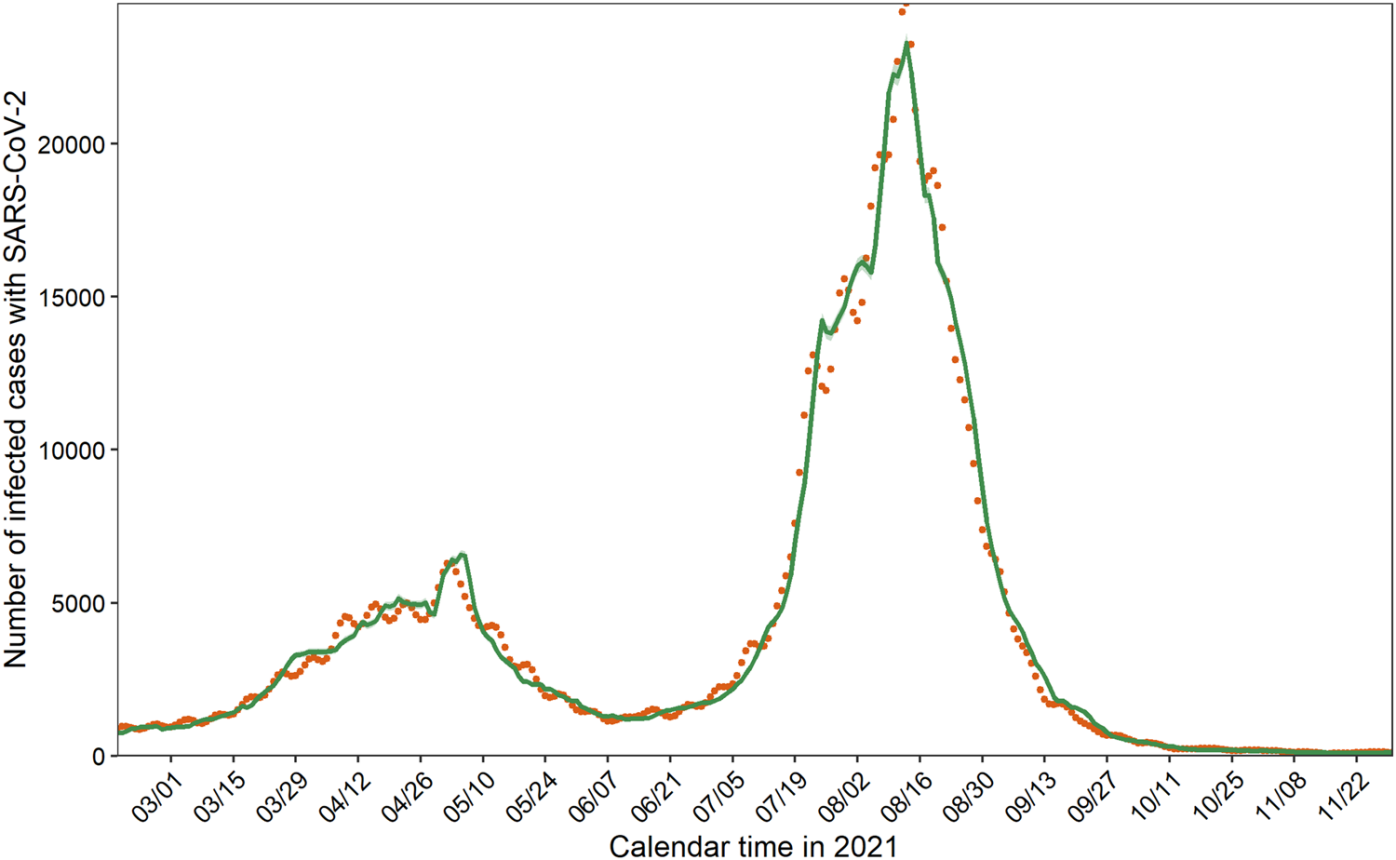
Comparison between predicted and observed infections with SARS-CoV-2. Orange dots represent the observed daily incidence of infection with SARS-CoV-2 during the primary series of the vaccination program from 17 February to 30 November 2021. Green line denotes the predicted daily incidence, computed by the transmission model, with 95% confidence intervals highlighted as light green areas. The observed number of COVID-19 cases is the same as the confirmed cases in this figure (i.e., we assumed that no ascertainment bias existed); in the main study, we examined realistic ranges of ascertainment bias.

Addressing age-dependent heterogeneity along with vaccination coverage, our transmission model successfully captured the observed data during the primary series of the vaccination program in Japan (Fig. 1 and Supplementary Fig. S8). Whereas the prototype model in Fig. 1 unrealistically assumed that observed cases represented all infected individuals (i.e., ascertainment bias factor at 1), hereinafter, we present results using other plausible reporting coverages, i.e., 0.125, 0.25, and 0.50, as shown in Supplementary Fig. S9 and Supplementary Table S1.

Hypothetical cumulative numbers of infections and deaths from February to November 2021 were explored in the absence of vaccination by different reporting coverages (Table 1). We found that the cumulative number of infections differed, from 63.3 million (95% CI 63.2–63.6) to 72.0 million (95% CI 71.4–72.6) cases for reporting coverages of 0.25 and 0.50, respectively. The possible cumulative number of deaths without vaccination ranged from 213,000 (95% CI 212–213) to 860,000 (95% CI 850–869) deaths for reporting coverage from 0.125 to 0.50. Compared with variations in cases, variations in deaths were broader because the infection fatality risk also varied by reporting coverage (Supplementary Figs. S10 and S11).

**Table 1:** Cumulative numbers of infections and deaths owing to COVID-19 without vaccination according to reporting coverage.

Reporting coverage	Infections (thousand)	Deaths (thousand)
0.50	72,015 (71,406–72,621)^a^	860 (850–869)
0.25	63,344 (63,242–63,562)	364 (363–366)
0.125	71,457 (71,338–71,646)	213 (212–213)

^a^Values inside the parentheses represent 95% confidence intervals computed using the parametric bootstrap method.

**Figure 2:**
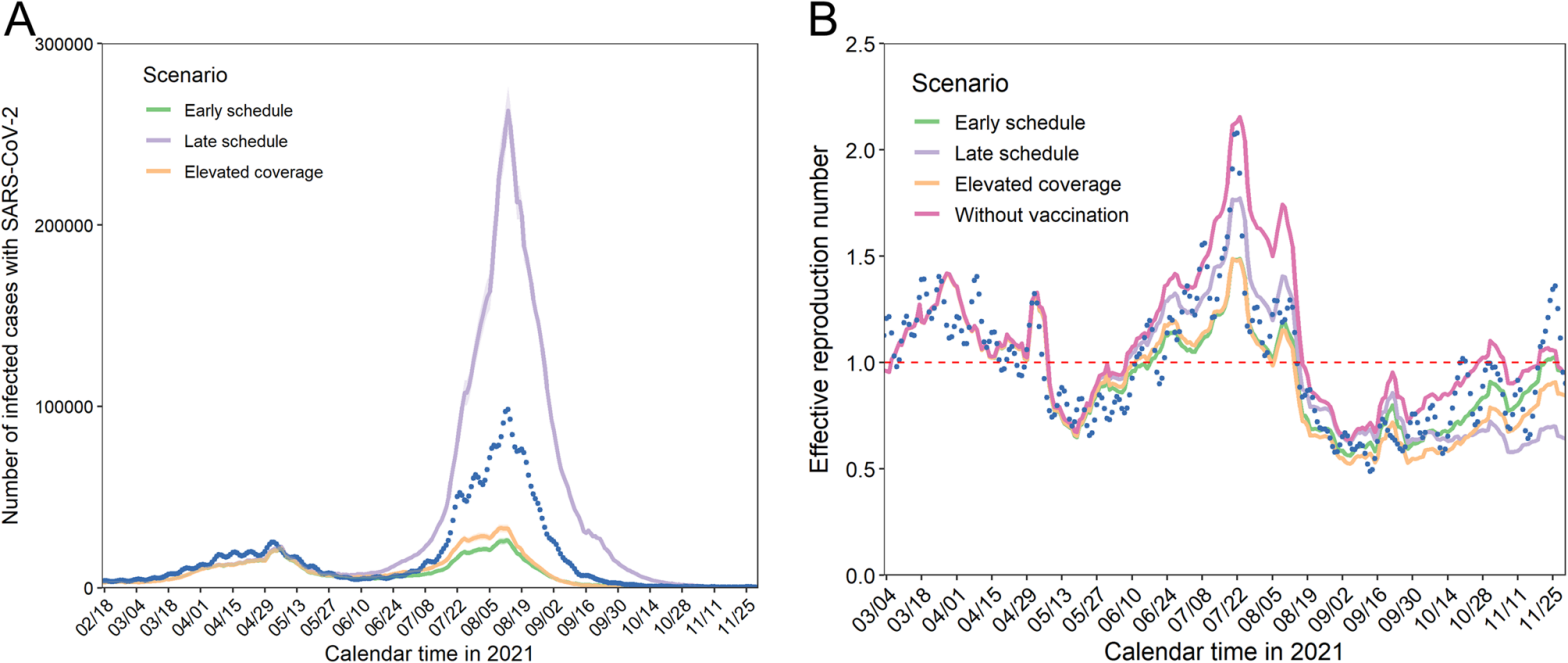
Impact of the primary series of the vaccination program on cases and the effective reproduction number. (**A**) Number of infections with SARS-CoV-2 from 17 February to 30 November 2021 according to counterfactual vaccination scenarios. Each line represents a different scenario with 95% confidence intervals highlighted as the light colored area; blue dots denote actual numbers of infections. (**B**) Effective reproduction number by vaccination scenario from 4 March to 30 November 2021. The colors are the same as in Fig. 2A. Blue dots represent the effective reproduction number estimated using the actual estimated infections shown in Fig. 2A. The pink-colored line represents the counterfactual scenario without vaccination. The red dashed line describes the threshold of the effective reproduction number, which is equal to 1. The number of infections was calculated assuming that the reporting coverage is 0.25.

Here, we present the results based on the assumption that the reporting coverage was 0.25, i.e., the actual number of infections was four times greater than observed (confirmed) cases^22^. The epidemic size varied greatly with the counterfactual vaccination scenario (Fig. 2A). If the vaccination program had been conducted 14 days earlier than the actual pace, the peak of daily incidence would have decreased by 73%, i.e., 98,368 infections (or four times the observed) versus 26,149 (95% CI 24,354–27,952) infections in the early schedule scenario. However, if the program had taken place 14 days later than the actual schedule, the peak of daily incidence would have reached 263,220 (95% CI 250,387–276,173) infections, and the maximum daily incidence was estimated to be 33,004 (95% CI 30,996–35,258) infections in the elevated coverage scenario. Using the estimated number of infections over time, we calculated the effective reproduction number, interpreted as the average number of infections generated by a single primary case at a certain time (Fig. 2B). We also computed the line representing the effective reproduction number in the scenario without vaccination. The discrepancies among scenarios became recognizable when the vaccination program was accelerated around June–July 2021, sharing similar incidence patterns (Fig. 2A). Comparing Fig. 2A and B, the peak height of the effective reproduction number did not necessarily correspond to the magnitude of the epidemic.

Table 2 presents the cumulative number of infections with SARS-CoV-2 by age group and counterfactual scenario. Whereas the early schedule and elevated coverage scenarios respectively could have contributed to reductions of 54% and 47% overall, the late schedule scenario could have led to an increase in infections of 117%, reaching more than 10 million infections by the end of November 2021. In all examined scenarios, young adults aged 20–29 years yielded the greatest number of infections whereas the relative and absolute reductions with better vaccination programs than the actual program were comparable among people aged 10–49 years.

**Table 2:** Cumulative numbers of infections with SARS-CoV-2 in the counterfactual scenarios.

Age group (years)	Scenario^a^	Estimate (thousand)	95% confidence interval	Relative change (%)^b^
0–9	Early	93	87–99	− 72
	Late	498	477–518	50
	Elevated	109	103–115	− 67
10–19	Early	324	304–345	− 30
	Late	1677	1611–1740	260
	Elevated	375	354–398	− 20
20–29	Early	537	508–567	− 56
	Late	2470	2373–2563	101
	Elevated	597	565–629	− 51
30–39	Early	314	295–332	− 60
	Late	1545	1478–1610	95
	Elevated	356	336–376	− 55
40–49	Early	344	324–364	− 53
	Late	1731	1654–1804	134
	Elevated	391	369–413	− 47
50–59	Early	266	251–281	− 54
	Late	1303	1243–1361	126
	Elevated	303	287–320	− 48
60–69	Early	120	114–127	− 52
	Late	495	470–518	98
	Elevated	148	140–156	− 41
70–79	Early	95	90–100	− 43
	Late	286	272–299	71
	Elevated	110	104–116	− 34
80–89	Early	57	54–61	− 47
	Late	148	141–154	36
	Elevated	64	61–68	− 41
≥ 90	Early	16	15–17	− 63
	Late	43	41–45	2
	Elevated	18	17–19	− 58
Total	Early	2166	2044–2290	-54
	Late	10,196	9761–10,607	117
	Elevated	2470	2337–2610	− 47

^a^Early: counterfactual scenario of a vaccination program implemented earlier that the actual schedule; Late: counterfactual scenario of a vaccination program implemented later that the actual schedule; Elevated: counterfactual scenario if the program had been implemented faster with higher vaccination coverage among adolescents and people aged 10–59 years.

^b^Relative change represents a comparison between the computed number and observed number (i.e., reporting coverage = 0.25).

The cumulative numbers of deaths by age group and counterfactual scenario are summarized in Table 3. Mortality in older people was more sensitive to different vaccination scenarios. In the late schedule scenario, the relative increase in the number of deaths was estimated to be 50%, i.e., this scenario yielded more than 5000 additional deaths by the end of November 2021.

**Table 3:** Cumulative numbers of deaths associated with COVID-19 in the counterfactual scenarios.

Age group (years)	Scenario^a^	Estimate (persons)	95% confidence interval	Relative change (%)^b^
0–9	Early	–	–	–
	Late	–	–	–
	Elevated	–	–	–
10–19	Early	2	2–2	− 31
	Late	11	10–11	260
	Elevated	2	2–3	− 19
20–29	Early	10	9–11	− 56
	Late	46	44–49	102
	Elevated	11	10–12	− 51
30–39	Early	27	24–29	− 60
	Late	131	124–138	95
	Elevated	30	28–33	− 55
40–49	Early	106	97–115	− 53
	Late	533	504–562	135
	Elevated	122	113–131	− 46
50–59	Early	301	276–326	− 54
	Late	1465	1381–1547	126
	Elevated	348	320–376	− 46
60–69	Early	533	480–587	− 49
	Late	1998	1859–2138	92
	Elevated	663	602–727	− 36
70–79	Early	1445	1296–1601	− 40
	Late	4000	3684–4325	67
	Elevated	1700	1532–1877	− 29
80–89	Early	2135	1874–2410	− 44
	Late	5089	4608–5586	34
	Elevated	2444	2156–2748	− 36
≥ 90	Early	717	565–880	− 61
	Late	1831	1558–2119	− 1
	Elevated	829	661–1009	− 55
Total	Early	5274	4623–5960	− 48
	Late	15,103	13,771–16,474	50
	Elevated	6150	5424–6916	− 39

^a^Early: counterfactual scenario of a vaccination program implemented earlier that the actual schedule; Late: counterfactual scenario of a vaccination program implemented later that the actual schedule; Elevated: counterfactual scenario if the program had been implemented faster with higher vaccination coverage among adolescents and people aged 10–59 years.

^b^Relative change represents a comparison between the computed number and observed number (i.e., reporting coverage = 0.25).

## Discussion

Whereas Japan successfully implemented its primary series of vaccination against COVID-19, reaching 75% coverage by the end of November 2021^23^, a pressing question has been how successful the program was during the pre-Omicron period. The present study revealed that without the vaccination program, the cumulative numbers of infections and deaths would have been 63.3 million (95% CI 63.2–63.6) and 364,000 (95% CI 363–366), respectively, assuming that confirmed cases represented 25% of infections. Despite the immense impact of the program, had vaccination been implemented 14 days earlier, there could have been 54% and 48% fewer cases and deaths, respectively, than the observed numbers. These figures represent the averted number of cases and deaths, and such estimates contrast to vaccine effectiveness (or efficacy) estimate at an individual level via randomized controlled trial or cohort study design, i.e., the averted number estimates require the vaccination coverage at the population level (possibly in real time), and additional datasets, including transmission dynamics, need to be analyzed to clarify the indirect effect of vaccination. The use of renewal process models enabled us to demonstrate the critical importance of the pace of the vaccination program and the prioritizing of vaccine recipients in determining the disease burden associated with COVID-19.

A critical take-home message from the present study is that the indirect effect of vaccination was enormous in Japan. The numbers of prevented infections and deaths were 13.5 and 36.4 times the empirically observed counts, respectively. In other words, the total effectiveness of the vaccination program in preventing infection and death was estimated at 92.6% and 97.2%, respectively. Of these fractions, the direct effect (i.e., comparison of risks between vaccinated and unvaccinated cases) that we estimated earlier^17^ accounted for only 3.6% and 5.1%, respectively, and the indirect effect (i.e., comparison of risks between actual and counterfactual courses of the epidemic) was as large as 89.0% and 92.1% reductions in infections and deaths, respectively. Such differences were seen because the incidence in Japan remained relatively lower than those in Western countries owing to PHSM, e.g., less than 5% of the population was reported as a COVID-19 case by the end of 2021. Clarifying the total effectiveness of vaccination was facilitated by modeling to yield the counterfactual scenario, and our finding regarding the total effect echoes those of published studies^18,19^. Together with past evidence^18,19^, consistent findings that the vaccination program prevented infections among half of the Japanese population and more than 90% of prevented deaths were owing to its indirect effect indicate that the vaccination program was enormously successful during the Delta variant epidemic wave during 2021 in Japan. The importance of indirect effect is what the present study contrasts to existing published studies^12–17^ that only directly measured individual benefit of vaccination, including the averted cases, hospitalization, severe complication and death. In many countries with greater incidence, including Brazil^15^, Israel^14^, Italy^12^, United States^16^ and countries that belong to WHO European Region^13^, the direct effect was already enormous. Japan enjoyed smaller incidence by the end of 2021 and the direct effect was relatively limited^17^, but the present study has been unique in that it demonstrated that the indirect effect can be inferred to be substantial using the effective reproduction number in a counterfactual scenario.

Another notable finding of this study is that our modeling approach enabled us to examine hypothetical scenarios in which the vaccination pace is accelerated. The cumulative numbers of infections in the early schedule and late schedule scenarios were estimated to be 2.2 million (95% CI 2.0–2.3) and 10.2 million (95% CI 9.8–10.6), respectively, which clearly led to substantial differences in mortality. Epidemiological studies can help policy makers recognize that a 1- or 2-week difference in the implementation of vaccination could yield completely different population impacts.

Published studies have indicated that prioritized vaccination for older people could minimize COVID-19 mortality if vaccines are not sufficiently available^24–28^. This was consistent with our finding, i.e., the early schedule scenario yielded better outcomes than the elevated coverage scenario. However, in our elevated coverage scenario (i.e., encouraging more adolescents and people aged 10–59 years to be vaccinated), the total effect was substantial, even when older people were not prioritized for vaccination. This demonstrates that vaccinating younger individuals with substantial transmission potential is a critical strategy in mitigating the magnitude of the epidemic for an entire population, including children aged < 10 years who were not eligible for the vaccination program. Taken together, the present study findings imply that, given a substantial vaccine supply and immunization capacity, allocating vaccines for younger adults in addition to prioritizing older adults could reduce the overall COVID-19 burden, as previously indicated^19,27,29–32^.

So, how should we rate the vaccination program in Japan during the SARS-CoV-2 Delta variant epidemic wave? The Japanese government set a goal for the daily number of vaccinated people of one million in early May 2021 (which was achieved from late June to July), subsequently stating that the maximum number was to be 1.5 million in the later part of the same month^33^. In addition to mass vaccination with initial prioritization of older people and health care workers, the program of vaccination in the workplace aimed at expanding coverage started in late June 2021^34^. Our counterfactual scenario indicated that the observed vaccination program helped avoid the worst case. However, if the vaccination program had begun 2 weeks later than the observed schedule, substantial mortality could have occurred. Additionally, a surge in COVID-19 patients observed in July–September 2021 was the largest epidemic wave ever experienced in Japan, and the corresponding period fell under the state of emergency, which was based on a non-legally binding policy in which the government requested voluntary restriction of contacts^35^. Were PHSM not in place under the state of emergency, the number of infections could have been even greater than the observed number. Considering that our early schedule and elevated coverage schedule scenarios were realistic in their anticipated pace of vaccination, considerable mortality and resulting economic losses could have been mitigated. Perhaps more importantly, from a scientific point of view, evidence regarding the indirect impact of such interventions in real time using modeling techniques should be routinely accessible to policy makers during future pandemics.

Our study involved several technical limitations. First, as previously mentioned, during the research period, Japan experienced three state of emergency declarations: from 8 January to 21 March, from 25 April to 20 June, and from 12 July to 30 September 2021. Rather than incorporating the specific variable of a state of emergency into the model (e.g., quantified effectiveness of PHSM), we tried to indirectly capture its impact via estimating the effective reproduction number using several explanatory variables, including mobility. In fact, use of human mobility data as a predictor is recognized as reflecting the impact of PHSM^36–39^. It should be noted, however, that published studies have attempted to measure the population-level impact of both vaccination and PHSM over the course of time^40,41^. Second, the contact matrix used in the present study was quantified before the study period^42^, and the next-generation matrix was calibrated during the course of the pandemic. At minimum, our time-dependent reproduction number helped capture the transmission dynamics over time and across ages (Fig. 1). Third, vaccine-induced immunity and immunity following natural infection were dealt with independently in the present study, and we did not account for the effect of waning immunity with the latter during the study period. We focused on the period shortly after vaccination and before the vaccination rollout, when approximately only 1% of the population experienced COVID-19 infection. Fourth, we did not take into account the heterogeneities over geographical space. Strictly speaking, the state of emergency covered different durations and areas according to prefecture, leading to specific variations in mobility information^43^. Finally, while vaccines against the Omicron variant, which is antigenically distinct, have shown reduced effectiveness compared to previously circulating variants^44,45^, the population-level impact of those changes have yet to be understood well^46^. In line with this, we have yet to understand whether the indirect effects of vaccination continued to accumulate and played a pivotal role in responding to the Omicron variant and its subvariants, including XBB. Future studies should address the issue of population impact during Omicron era.

## Conclusions

We demonstrated that the indirect effect of vaccination in Japan during 2021 was very large, with the vaccination program reducing mortality by more than 97%. The pace of vaccination and prioritization of vaccine recipients have been key to mitigating the mortality burden of COVID-19. In the future, firm and prompt policy-making process based on real-time understanding of the transmission dynamics under various vaccination scenarios is called for.

## Methods

### Conversion to infections

COVID-19 was designated a notifiable disease under the infectious disease law of Japan as of 2021. All individuals suspected of being infected with SARS-CoV-2 were tested via PCR or quantitative antigen test at medical facilities. They were then requested to remain in home isolation and undergo investigation by municipal public health centers to identify their close contacts. Information of confirmed cases (e.g., age and sex) was registered in the Health Center Real-time Information-sharing System on COVID-19 (HER-SYS) by medical facilities or municipal public health centers. Supplementary Fig. S1A shows the number of confirmed cases from the beginning of the primary series (the first and second doses) of the vaccination program through the end of November 2021. In the end of November 2021, SARS-CoV-2 in Japan was dominated by Delta variant to which the vaccine effectiveness was known to have been greatly diminished, sometimes by 10%, compared with other variants that circulated earlier^47–49^.

The time of infection for all confirmed COVID-19 cases retrieved from HER-SYS was backcalculated using a previously estimated distribution of the interval between infection and illness onset, assumed to follow a log-normal distribution with a mean of 4.6 days and standard deviation (SD) of 1.8 days^50, 51^. Cases without a date of symptom onset were backcalculated using the time difference from symptom onset to reporting, assumed to follow a log-normal distribution with a mean of 2.6 days and SD of 2.1 days, as previously estimated using cases with information for the date of symptom onset. Non-parametric backcalculation was performed using the R-package “surveillance” (version 1.20.3). To address the issue of reporting bias, we explored different reporting coverages: 0.125, 0.25, 0.5, and 1.0 (no bias) by multiplying the backcalculated cases by 1 and dividing by reporting coverage to finally obtain the number of infections.

### Immune fraction

SARS-CoV-2, all vaccinated individuals retrieved from the Vaccine Record System (VRS) were converted into immunized people according to time. The data comprised the sex, age, and date of vaccination for vaccinated individuals. We assumed that all people who received the first dose were subsequently vaccinated with the second dose at an interval of 21 days (Supplementary Fig. S1B). According to statistics of the VRS, there was a very small discrepancy in vaccination coverage between the first dose (75.19%) and the second dose (74.61%) as of the end of December 2021^52^; therefore, we could obtain a certain consensus on the usage data for people vaccinated with the first dose only. For the conversion, we used a profile of vaccine efficacy involving waning immunity for the primary series used by Gavish et al.^19^, which was based on previous estimates^53,54^. Given the widespread use of the messenger RNA vaccine BNT162b2 (Pfizer/BioNTech) in Japan (more than 80% of individuals received this vaccine by the end of November 2021)^23^, we assumed that published estimates could directly be applied to the case of Japan. Further details and background of the primary series in Japan’s vaccination program are described elsewhere^17^.

To adapt the following transmission model, we used the number of vaccinated individuals and the profile of vaccine efficacy to estimate the immune fraction in age group $a$ at calendar time $t$, ${l}_{a,t}$, which is expressed as:

1 $${l}_{a,t}=\frac{1}{{n}_{a}}\sum_{s=1}^{t-1}{v}_{a,t-s}{h}_{s}$$

where ${n}_{a}$ is the population size in age group $a$ in 2021^55^, ${v}_{a,t}$ denotes the number of vaccinated individuals in age group $a$ at calendar time $t$, and ${h}_{s}$ represents the vaccine profile. Supplementary Fig. S2 displays the estimated immune fraction by age group.

### Transmission model

We developed the time-dependent transmission model that accounts for heterogeneous transmission between age groups, fitting the model to observed incidence data and estimating unknown parameters. We used the following renewal equation to infer the transmission dynamics underlying the COVID-19 epidemic, which is described as:

2 $${i}_{a,t}=\sum_{b=1}^{10}\sum_{\tau =1}^{t-1}{{\varvec{R}}}_{{\varvec{a}}{\varvec{b}},{\varvec{t}}}{i}_{b,t-\tau }{g}_{\tau },$$

where ${i}_{a,t}$ represents the number of infections with SARS-CoV-2 in age group $a$ at day $t$ and ${g}_{\tau }$ indicates the probability density function of the generation interval, assumed to follow a Weibull distribution with a mean of 4.8 days and SD of 2.2 days^51,56^. ${{\varvec{R}}}_{{\varvec{a}}{\varvec{b}},{\varvec{t}}}$ denotes the effective reproduction number, interpreted as the average number of secondary cases in age group $a$ generated by a single primary case in age group $b$ at calendar time $t$. To capture the impact of vaccination, ${{\varvec{R}}}_{{\varvec{a}}{\varvec{b}},{\varvec{t}}}$ was decomposed as:

3 $${{\varvec{R}}}_{{\varvec{a}}{\varvec{b}},{\varvec{t}}}=\left(1-{l}_{a,t}-\frac{\sum_{k=1}^{t-1}{i}_{a,k}}{{n}_{a}}\right){{\varvec{K}}}_{{\varvec{a}}{\varvec{b}}}p{h}_{t}{d}_{t}{c}_{t}$$

where $\sum_{k=1}^{t-1}{i}_{a,k}$ represents the cumulative number of previous infections after 16 February 2021. ${{\varvec{K}}}_{{\varvec{a}}{\varvec{b}}}$ is considered a next-generation matrix, which was modeled as ${{\varvec{K}}}_{{\varvec{a}}{\varvec{b}}}={{{s}}}_{{{a}}}{{\varvec{m}}}_{{\varvec{a}}{\varvec{b}}}$, where ${{{s}}}_{{{a}}}$ represents relative susceptibility and ${{\varvec{m}}}_{{\varvec{a}}{\varvec{b}}}$ denotes the contact matrix; we rescaled a previously quantified next-generation matrix during the initial phase of the COVID-19 epidemic in 2021 attributable to the Alpha variant^57^. Because the oldest age group was ≥ 65 years in the previous estimate, we reconstructed the epidemic curve with new age groups: 0–9, 10–19, 20–29, 30–39, 40–49, 50–59, 60–69, 70–79, 80–89 and ≥ 90 years and estimated ${{\varvec{K}}}_{{\varvec{a}}{\varvec{b}}}$ by fitting the model to observed cases (Supplementary Fig. S3). The detailed methods are explained elsewhere^57,58^. We assumed that the contact rates among groups aged ≥ 70 years were the same as those aged ≥ 65 in the contact matrix, ${{\varvec{m}}}_{{\varvec{a}}{\varvec{b}}}$, which is based on a social epidemiological survey conducted prior to the COVID-19 pandemic in Japan^42^. With respect to the above explanation, those early terms in Eq. (3) could capture the effective heterogeneous interactions between infectees and infectors, which accounts for the immune fraction owing to vaccination and infections among susceptible individuals (i.e., infectees). $p$ denotes the scaling parameter involving all terms in Eq. (3) and ${h}_{t}$ expresses the change in mobility. The variable, ${h}_{t}$, related to human mobility was decomposed as:

4 $${h}_{t}={\omega }^{community}{\alpha }_{t}^{community}+{\omega }^{house}{\alpha }_{t}^{house}+{\omega }^{work}{\alpha }_{t}^{work},$$

where $\omega$ means the coefficient of human mobility in the community, household, or workplace relative to the community setting (i.e., ${\omega }^{community}$ is equivalent to 1). The coefficient, ${\alpha }_{t}$, describes a proxy of the intensified contacts in three different settings retrieved from Google's COVID-19 community mobility report in Japan^59^. Those data were smoothed using a 7-day moving average (Supplementary Fig. S4). ${d}_{t}$ represents the increase in transmissibility of the Delta variant compared with earlier variants, which was formulated as ${d}_{t}=r{u}_{t}$, where $r$ is the scaling parameter for transmissibility and ${u}_{t}$ represents the profile of increased transmissibility. We assumed that ${u}_{t}$ increased with the detected proportion of COVID-19 cases owing to the Delta variant in Japan^60^, which was modeled using a logistic curve. We then rescaled ${u}_{t}$ up from 1 to a maximum of 1.5^61–63^. A comparison between the predicted and observed proportion is shown in Supplementary Fig. S5. ${d}_{t}$ was parameterized as 1 before 20 May 2021, when we assumed that the proportion of infections with the Delta variant started to increase at population level. Finally, ${c}_{t}$ expresses the influence of consecutive holidays, defined as more than 3 days in the present study. Moreover, we added “Obon season,” the national religious season associated with Buddhist tradition, to those holidays. Not all consecutive days in this period (from 13 to 16 August 2021) were regarded as holidays; however, many Japanese people travel and/or visit their relatives during this season. We modeled ${c}_{t}$ as ${c}_{t}=e{\beta }_{t}$, where $e$ accounts for the coefficient of holiday influence and ${\beta }_{t}$ was assigned 1 if the day was aligned with consecutive holidays; otherwise ${c}_{t}$ was parameterized as 1.

### Vaccination scenarios

We first computed the counterfactual scenario, i.e., without vaccination. We also explored three additional hypothetical scenarios: (1) the vaccination program was implemented sooner than the actual program, reaching a maximum number of vaccinated individuals 14 days earlier than the observed pace (hereafter “early schedule” scenario); (2) the vaccination schedule was delayed, reaching a peak in the number of vaccinated people 14 days slower than the observed pace (“late schedule” scenario); and (3) adolescents and people aged 10–59 years were vaccinated more and faster (“elevated” scenario). To explore different counterfactual scenarios, we first regressed the vaccination coverage using the logistic function by age group, which is modeled as:

7 $$E({v}_{a,t})=\frac{{\pi }^{1}}{1+\mathrm{exp}(-{\pi }^{2}(t-{\pi }^{3}))},$$

where ${\pi }^{1}$, ${\pi }^{2}$, and ${\pi }^{3}$ represent the carrying capacity (eventual coverage of the primary series), speed of increase in the vaccination coverage, and requisite duration for the half coverage of ${\pi }^{1}$ (also representing the peak day for the number of vaccinated individuals), respectively. We performed maximum likelihood estimation to estimate ${\pi }^{1}$, ${\pi }^{2}$, and ${\pi }^{3}$ by age group. Comparisons between the predicted and observed number of vaccinated people by age group are shown in Supplementary Fig. S6.

We assumed that the days with the maximum number of vaccinated people (i.e., days that 50% of the carrying capacity was achieved) were 14 days earlier in the Early scenario and later in the Late scenario than the observed. For the Elevated scenario, we assumed that people aged 10–59 years had earlier peaks in the number of vaccinated individuals, as with the Early scenario. Additionally, people aged 10–19 years and aged 20–49 years were assumed to reach 70% and 90% in eventual vaccination coverage (${\pi }^{1}$), respectively. People aged ≥ 50 years had already reached more than 90% of the vaccination coverage by the end of November 2021. We did not consider vaccination among individuals aged less than 10 years because children were not eligible to be vaccinated during the primary series of the program in Japan. All scenarios of the vaccination program by age group are shown in Supplementary Fig. S7.

### Likelihood function

We assumed that the daily counts of infections followed a Poisson distribution, and the likelihood function with unknown parameters, $\theta =\{p,{\omega }^{house},{\omega }^{work},r,e\}$, was represented as:

8 $$L\left(\theta ;{i}_{a,t}\right)=\prod_{t}\prod_{a}\frac{{E({i}_{a,t})}^{{i}_{a,t}}\mathrm{exp}(-E({i}_{a,t}))}{{i}_{a,t}!}$$

By minimizing the loglikelihood function, we estimated $\theta$. The 95% confidence intervals (CI) were calculated from 1000 bootstrap iterations using the multivariate normal distributions of the parameters. We estimated a series of parameters by reporting coverage in the present study. All estimated parameters with 95% CIs are shown in Supplementary Table S1. Supplementary Fig. S8 demonstrates the fitting outcome of the predicted and observed infections with SARS-CoV-2 by age group, with reporting coverage of 1 (i.e., no ascertainment bias). Supplementary Fig. S9 compares the predicted and observed infections by reporting coverage.

Using the estimated parameters, $\theta$, we explored hypothetical scenarios by varying the timing and the recipients of vaccination. For this, we used infections already backcalculated 14 days back from the start of vaccination as the initial condition.

### Effective reproduction number

Because the effective reproduction number in Japan conventionally uses an estimate for the entire population, we also calculated an effective reproduction number based on the total number of cases at calendar time $t$, ${R}_{t}$, in each counterfactual scenario using the total number of infections with SARS-CoV-2. Using an equation similar to Eq. (2), the total number of infections, ${i}_{t}^{total}$, was modeled as:

9 $${i}_{t}^{total}={R}_{t}\sum_{\tau = 1}^{t-1}{i}_{t-\tau}^{total}{g}_{\tau}.$$

Assuming the daily case counts followed a Poisson distribution, we estimated ${R}_{t}$ using maximum likelihood estimation^51^.

### Infection fatality risk

To compute the mortality impact, we estimated the age-specific infection fatality risk (IFR) according to reporting coverage in the present study. First, we formulated the cumulative number of deaths in age group $a$ resulting from cases infected during the research period in unvaccinated and vaccinated individuals, respectively, which are described as:

10 $$\left\{\begin{array}{c}{D}_{a}^{unvaccinated}={IFR}_{a}\sum_{t=17\mathrm{ Feb }2021}^{30\mathrm{ Nov }2021}(1-{\epsilon }_{a,t}){\widehat{i}}_{a,t}\\ {D}_{a}^{vaccinated}={IFR}_{a}(1-{VE}_{a})\sum_{t=17\mathrm{ Feb }2021}^{30\mathrm{ Nov }2021}{\epsilon }_{a,t}{\widehat{i}}_{a,t}\end{array}\right.,$$

where ${\epsilon }_{a,t}$ represents the time-varying proportion of vaccinated people among confirmed cases in age group $a$ at calendar time $t$, and ${\widehat{i}}_{a,t}$ is the expected number of infections estimated from the transmission model. ${VE}_{a}$ expresses the vaccine-induced reduction in mortality estimated in 2021 for Japan^64^. We obtained ${\epsilon }_{a,t}$ by modeling cases with a vaccination history registered in HER-SYS using a logistic function. The observed proportion was calculated as 7-day moving average and shifted − 5 days because of the conversion for the time of infection. Also, to account for the age groups used in the present study, people aged 10–19, 20–29, 30–39, 40–49, 50–59 and ≥ 60 years were utilized as people aged 15–24, 25–34, 35–44, 45–54, 55–64 and ≥ 65 years for the proportion retrieved from HER-SYS, respectively. Supplementary Fig. S10 shows the comparison between the model prediction and observed proportions.

To estimate IFR by age group, the following likelihood equation was used:

11 $$L\left({\lambda }_{a};{\widehat{i}}_{a,t},{D}_{a}\right)=\prod \left(\begin{array}{c}\sum {\widehat{i}}_{a,t}\\ {D}_{a}\end{array}\right){\lambda }_{a}^{{D}_{a}}{\left(1-{\lambda }_{a}^{{D}_{a}}\right)}^{\sum {\widehat{i}}_{a,t}{-D}_{a}},$$

where ${\lambda }_{a}$ denotes the risk of death in age group $a$, modeled as:

12 $${\lambda }_{a}=\frac{({D}_{a}^{unvaccinated}+{D}_{a}^{vaccinated})}{\sum {\widehat{i}}_{a,t}}.$$

${D}_{a}$ is the cumulative number of deaths reported from 10 March to 21 December 2021 in age group $a$, which was retrieved from the Ministry of Health, Labour and Welfare of Japan, accounting for the reporting delay of 21 days.^65^ By minimizing the negative logarithm of Eq. (8), we estimated ${IFR}_{a}$. We performed this process for each reporting coverage. Supplementary Fig. S11 displays the estimated IFR by reporting coverage and age group. Finally, we estimated the cumulative number of deaths as an aggregation of ${D}_{a}^{unvaccinated}$ and ${D}_{a}^{vaccinated}$ in Eq. (7) according to different counterfactual scenarios of varying ${\widehat{i}}_{a,t}$. We only applied the first equation in Eq. (7), i.e., ${D}_{a}^{unvaccinated}$, for the counterfactual scenario in the absence of vaccination.

For calculation of the death toll, we altered only a parameter representing the requisite duration for the half coverage of a carrying capacity to coincide with changes in vaccine recipients in the counterfactual vaccination scenarios. Because of this exercise, we were able to model the specific proportion of vaccinated people among confirmed cases according to different vaccination scenarios. The principal idea of the logistic model is explained in the early subsection.

### Ethical considerations

This study was conducted according to the principles of the Declaration of Helsinki. Informed consent was obtained for reporting the diagnosis. The authors did not have an access to any individual identity information, and this research was approved by the Ethics Committee of Kyoto University Graduate School of Medicine (approval number R2673).

## Supplementary Information

Supplementary Information 1.

Supplementary Information 2.

Supplementary Information 3.

## Data Availability

We were allowed to access the information on HER-SYS only for the purpose of analyzing the COVID-19 situation in Japan; therefore, this database is not publicly available. However, we shared the daily numbers of vaccinated individuals and reported cases not stratified by age group during the analysis. Hiroshi Nishiura should be contacted to request the data from this study.
