# Author Correction: Evaluating the COVID‑19 vaccination program in Japan, 2021 using the counterfactual reproduction number

**DOI:** 10.1038/s41598-025-14940-x

**Published:** 2025-08-18

**Authors:** Taishi Kayano, Yura Ko, Kanako Otani, Tetsuro Kobayashi, Motoi Suzuki, Hiroshi Nishiura

**Affiliations:** 1 Kyoto University School of Public Health, Yoshida‑Konoe‑cho, Sakyo‑ku, Kyoto, 606‑8501 Japan; 2 Center for Surveillance, Immunization, and Epidemiologic Research, National Institute of Infectious Diseases, Tokyo, 162‑8640 Japan; 3 Department of Virology, Tohoku University Graduate School of Medicine, Miyagi, 980‑8575 Japan

Correction to: *Scientifc Reports* 10.1038/s41598-023-44942-6, published online 18 October 2023

The original version of this Article contained errors in the Data availability section. The raw epidemiological data used in the study is not publicly available but can be accessed through an application to the National Institute of Infectious Diseases, Japan Institute for Health Security.

As a result, in the Data availability section,

“Hiroshi Nishiura should be contacted to request the data from this study.”

now reads:

“Access to the raw epidemiological data used in the analysis can be requested through an application to the National Institute of Infectious Diseases, Japan Institute for Health Security at https://www.mhlw.go.jp/stf/seisakunitsuite/bunya/kenkou_iryou/kenkou/kekkaku-kansenshou/idb_index.html”

In addition, the Code availability section was omitted. The authors have shared the code through Zenodo.

The Code availability section was added and reads:

“The code developed to generate the results of the study is available at https://zenodo.org/records/15244462”

The original Article has been corrected.

