# Correction: Evaluating the COVID-19 vaccination program in Japan, 2021 using the counterfactual reproduction number

**DOI:** 10.1038/s41598-025-21251-8

**Published:** 2025-10-28

**Authors:** Taishi Kayano, Yura Ko, Kanako Otani, Tetsuro Kobayashi, Motoi Suzuki, Hiroshi Nishiura

**Affiliations:** 1 Kyoto University School of Public Health, Yoshida‑Konoe‑cho, Sakyo‑ku, Kyoto 606‑8501 Japan; 2 Center for Surveillance, Immunization, and Epidemiologic Research, National Institute of Infectious Diseases, Tokyo, 162‑8640 Japan; 3 Department of Virology, Tohoku University Graduate School of Medicine, Miyagi, 980‑8575 Japan

Correction to: *Scientific Reports* 10.1038/s41598-023-44942-6, published online 18 October 2023

The original version of this Article contained errors.

As a result of an error during figure preparation, the caption of Figure 2 was incorrect. As a result, in the Results section:

“Impact of the primary series of the vaccination program on cases and the effective reproduction number. (A) Number of infections with SARS-CoV-2 from 17 February to 30 November 2021 according to counterfactual vaccination scenarios. Each line represents a different scenario with 95% confidence intervals highlighted as the light colored area; blue dots denote actual numbers of infections. (B) Effective reproduction number by vaccination scenario from 4 March to 30 November 2021. The colors are the same as in Fig. 3A. Blue dots represent the effective reproduction number estimated using the actual estimated infections shown in Fig. 3A. The pink-colored line represents the counterfactual scenario without vaccination. The red dashed line describes the threshold of the effective reproduction number, which is equal to 1. The number of infections was calculated assuming that the reporting coverage is 0.25.”

now reads:

“Impact of the primary series of the vaccination program on cases and the effective reproduction number. (A) Number of infections with SARS-CoV-2 from 17 February to 30 November 2021 according to counterfactual vaccination scenarios. Each line represents a different scenario with 95% confidence intervals highlighted as the light colored area; blue dots denote actual numbers of infections. (B) Effective reproduction number by vaccination scenario from 4 March to 30 November 2021. The colors are the same as in Fig. 2A. Blue dots represent the effective reproduction number estimated using the actual estimated infections shown in Fig. 2A. The pink-colored line represents the counterfactual scenario without vaccination. The red dashed line describes the threshold of the effective reproduction number, which is equal to 1. The number of infections was calculated assuming that the reporting coverage is 0.25.”

In addition, in the Methods section, under the subheading ‘Effective reproduction number’, Equation 9 was incorrectly given as

$$i_{t}^{total} = R_{t} \mathop \sum \limits_{\tau }^{1 - \tau } i_{t - \tau }^{total} g_{\tau } .$$

The correct Equation 9 appears below.

$$i_{t}^{total} = R_{t} \mathop \sum \limits_{\tau = 1}^{t - 1} i_{t - \tau }^{total} g_{\tau } .$$

The original Article was corrected.

