# Supplementary material for: Evaluating the COVID-19 vaccination program in Japan, 2021 using the counterfactual reproduction number
Source: Sci Rep. 2023 Oct 18;13:17762. doi: 10.1038/s41598-023-44942-6 (PMC10584853; doi:10.1038/s41598-023-44942-6)
Supplement: Supplementary file 1 — Supplementary Information 1. [file 41598_2023_44942_MOESM1_ESM.docx]

Supplementary Information

**Evaluating the COVID-19 vaccination program in Japan, 2021 using the counterfactual reproduction number**

**The file includes:**

Supplementary Figure S1 to S11

Supplementary Table S1


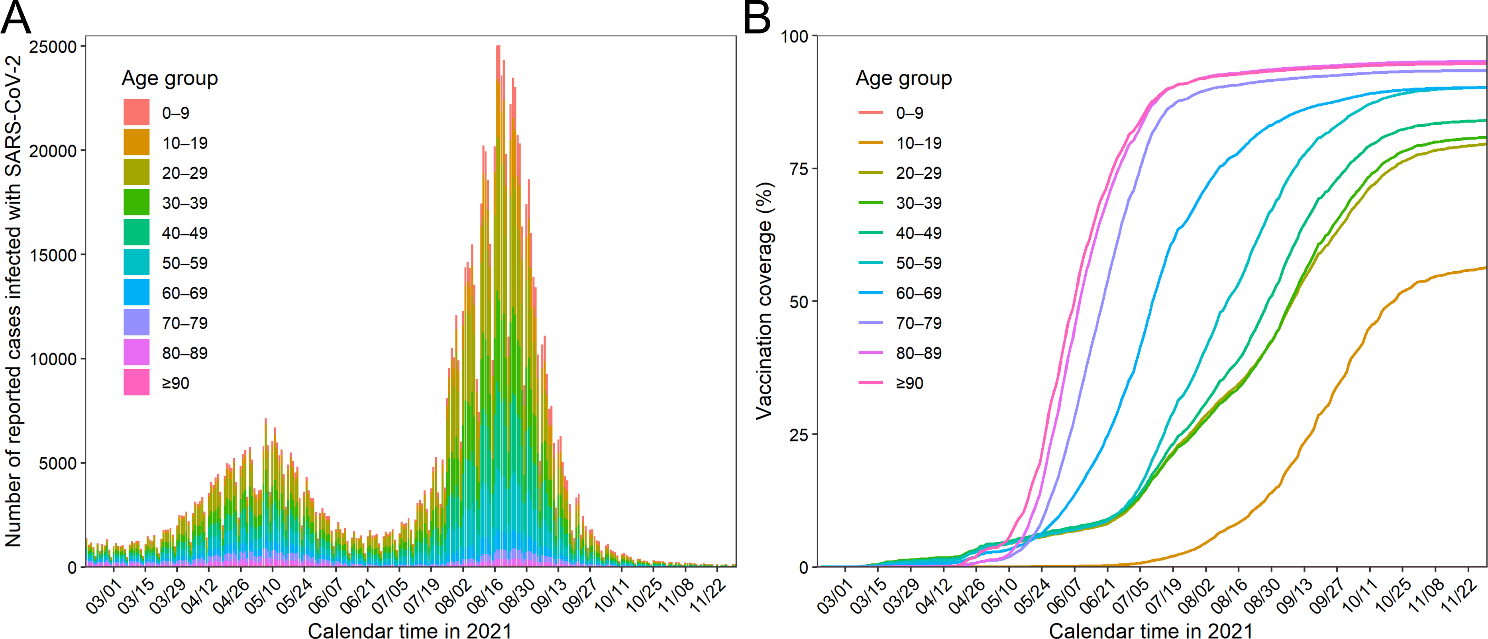


**Supplementary Figure S1. Epidemiological background during the primary series of the vaccination program in Japan.**

(A) Daily number of reported COVID-19 cases from 17 February to 30 November 2021 by age group. (B) Vaccination coverage of the first dose in the same period as Supplementary Figure 1A by age group.

**
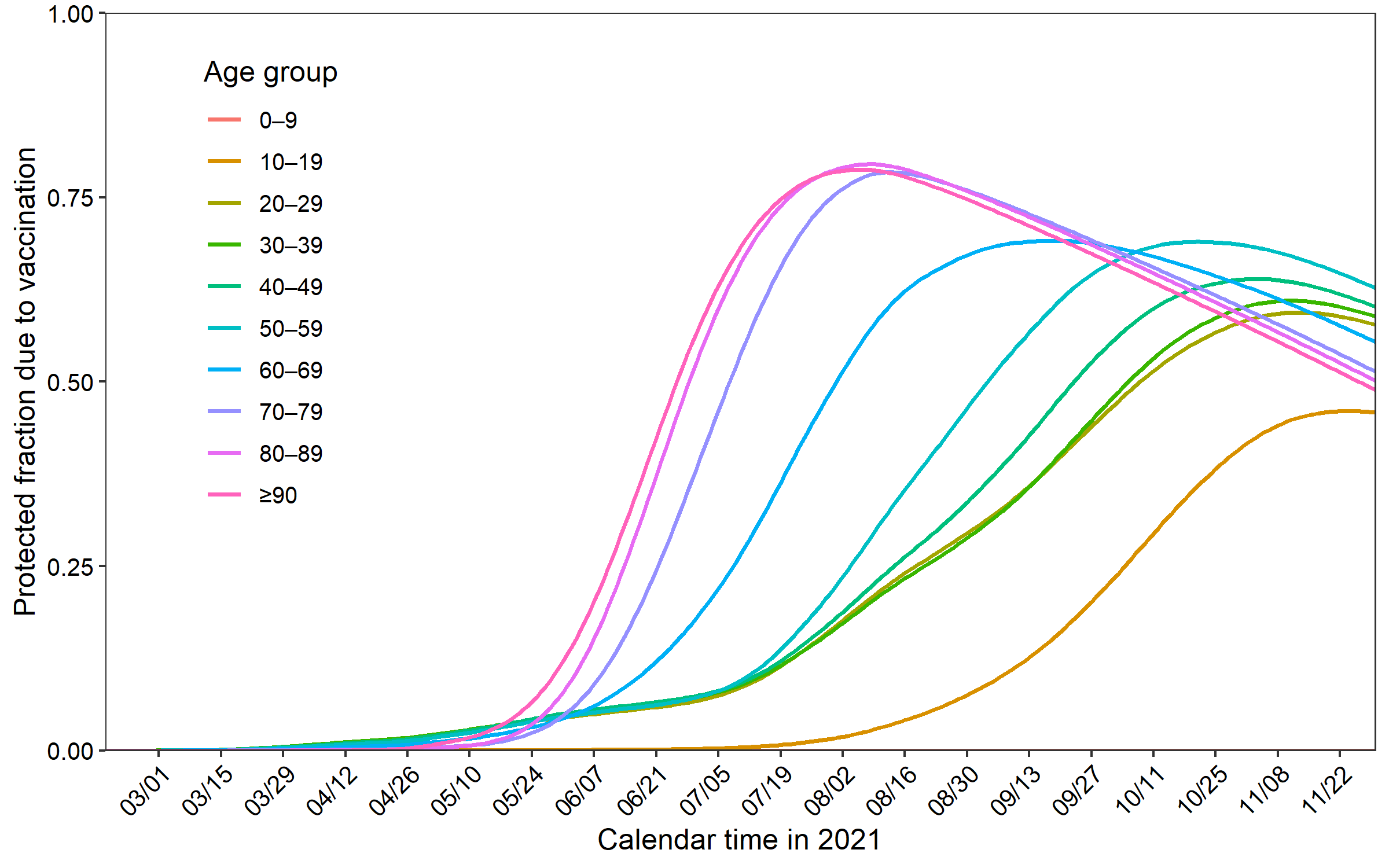
**

**Supplementary Figure S2. Immune fraction by age group.**


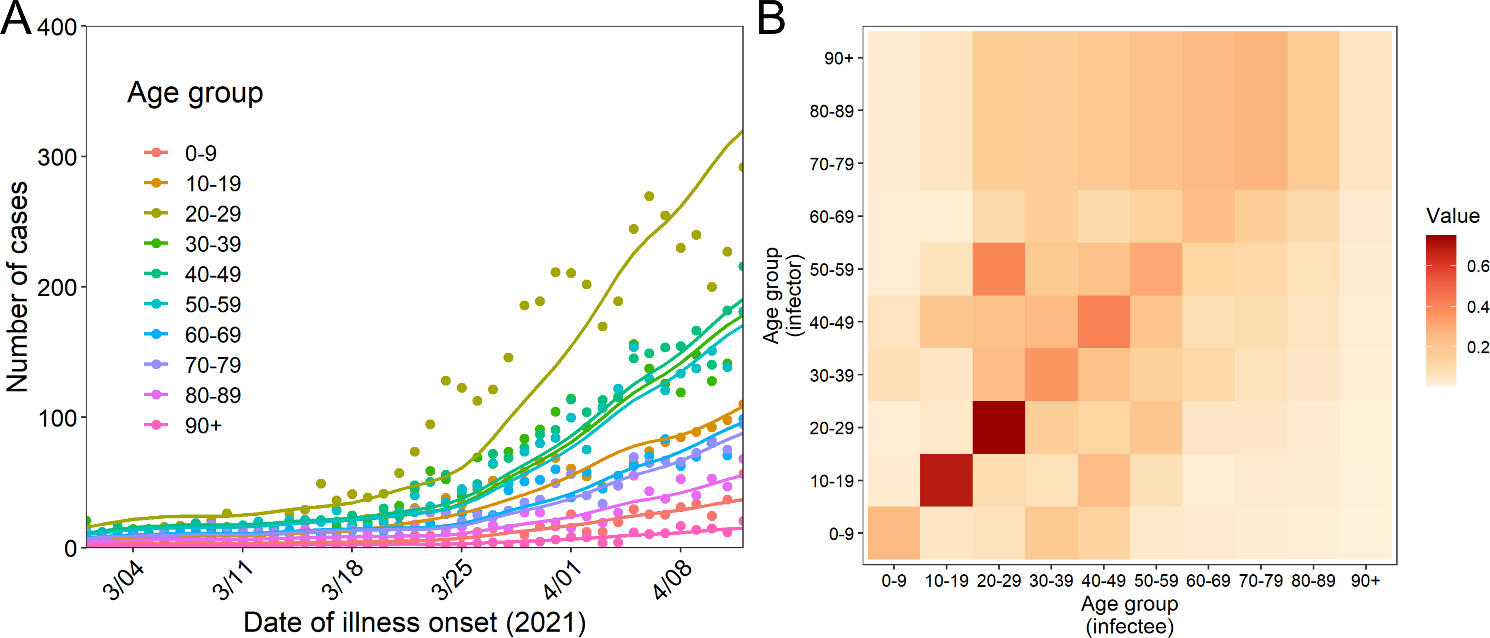


**Supplementary Figure S3. Next-generation matrix quantified in the initial phase of the epidemic wave involving the SARS-CoV-2 Alpha variant.**

(A) Comparisons between predicted and observed cases in Osaka, Japan. Colored dots represent observed cases, and lines illustrate the predicted cases from 1 March to 12 April 2021. (B) Next-generation matrix.


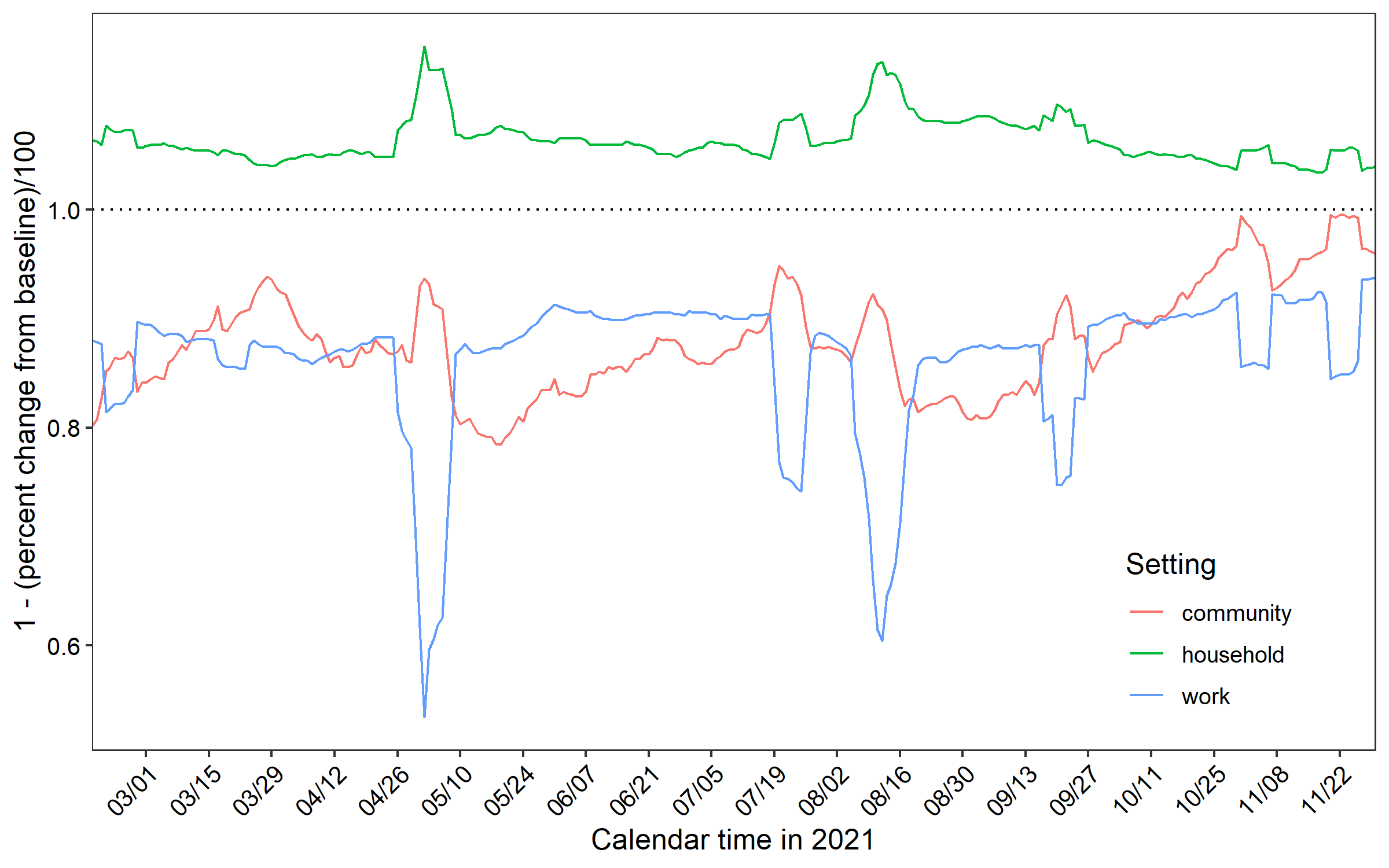


**Supplementary Figure S4. Daily coefficients of human mobility.**

The data were retrieved from Google Community Mobility Reports in Japan between 17 February and 30 November 2021. Daily coefficients are represented as percent change from baseline. The community, household, and workplace were based on percent change for retail and recreation, residential, and workplaces, respectively, in the Google reports.


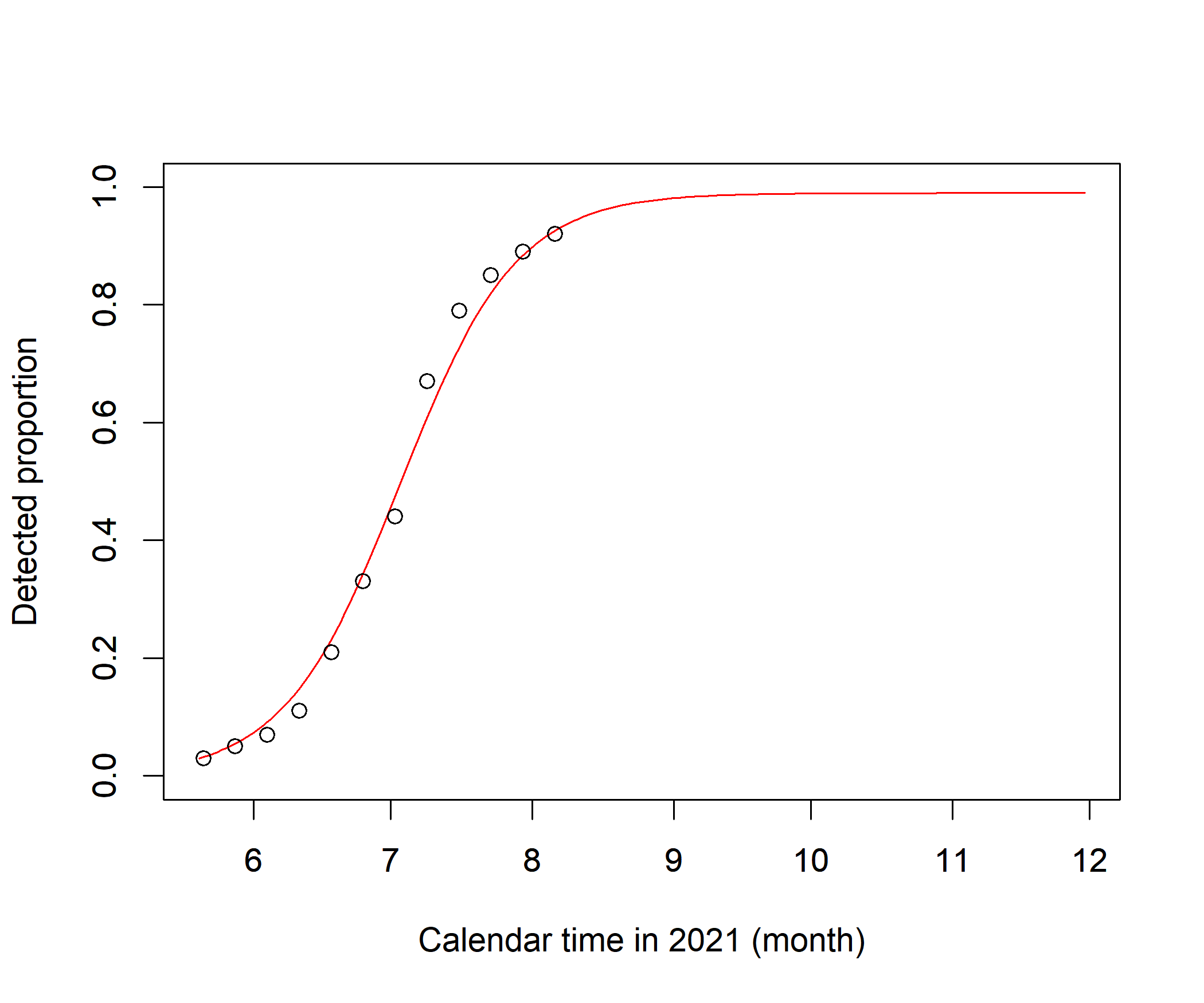


**Supplementary Figure S5. Comparison between predicted and observed proportion of detected cases involving the SARS-CoV-2 Delta variant.**

Each black dot represents the observed proportion of detected cases of infection with the Delta variant in Japan. Red curve denotes the predicted proportion of detected cases. The data were censored on week 34 (from 23 to 29 August 2021) when the detected proportion reached the maximum; we assumed this continued to increase during the research period.


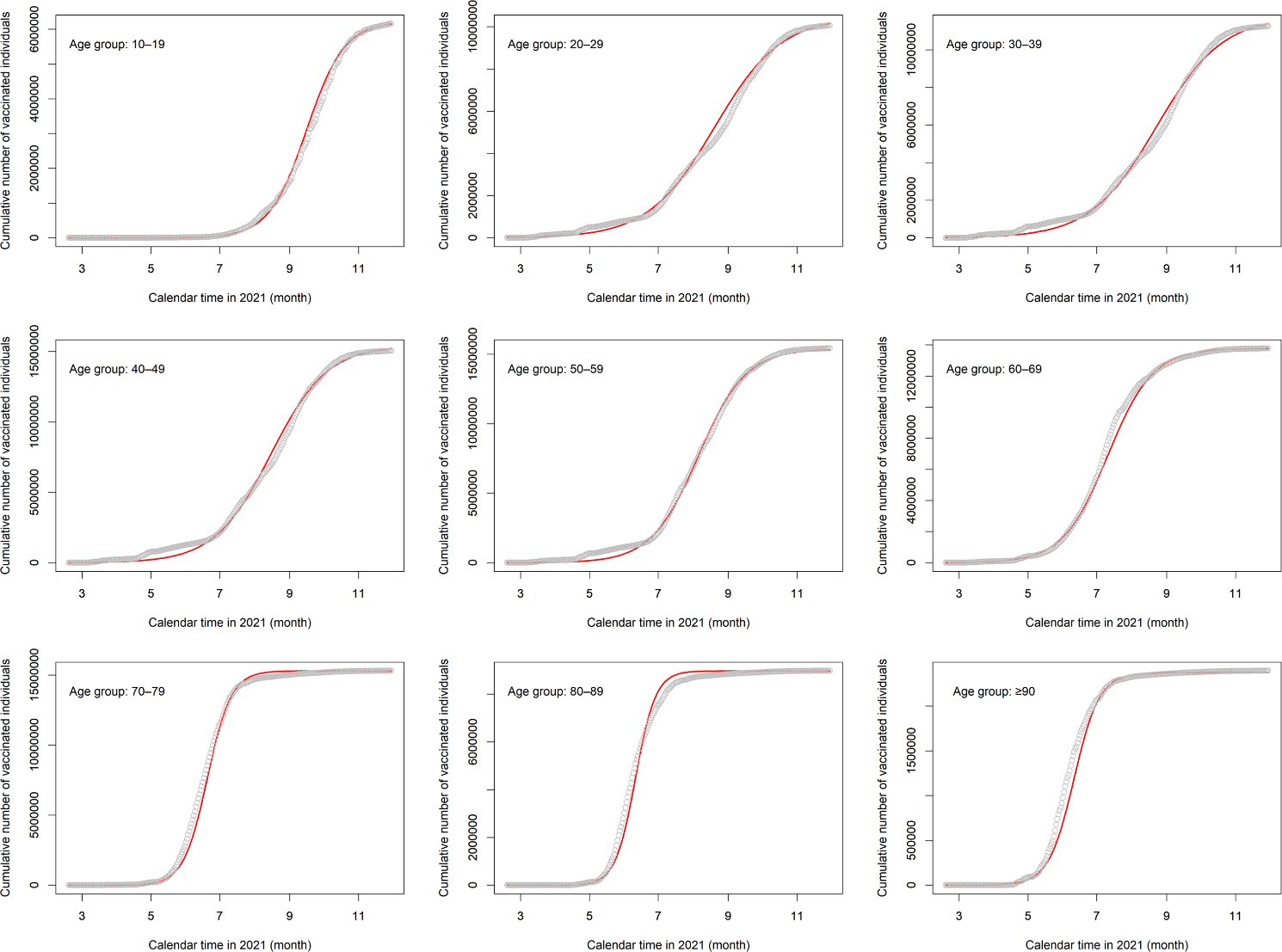


**Supplementary Figure S6. Comparison between predicted and observed number of vaccinated individuals by age group.**

Gray circles represent the observed numbers of vaccinated individuals. Red lines describe the numbers of vaccinated people predicted from the logistic function.

**
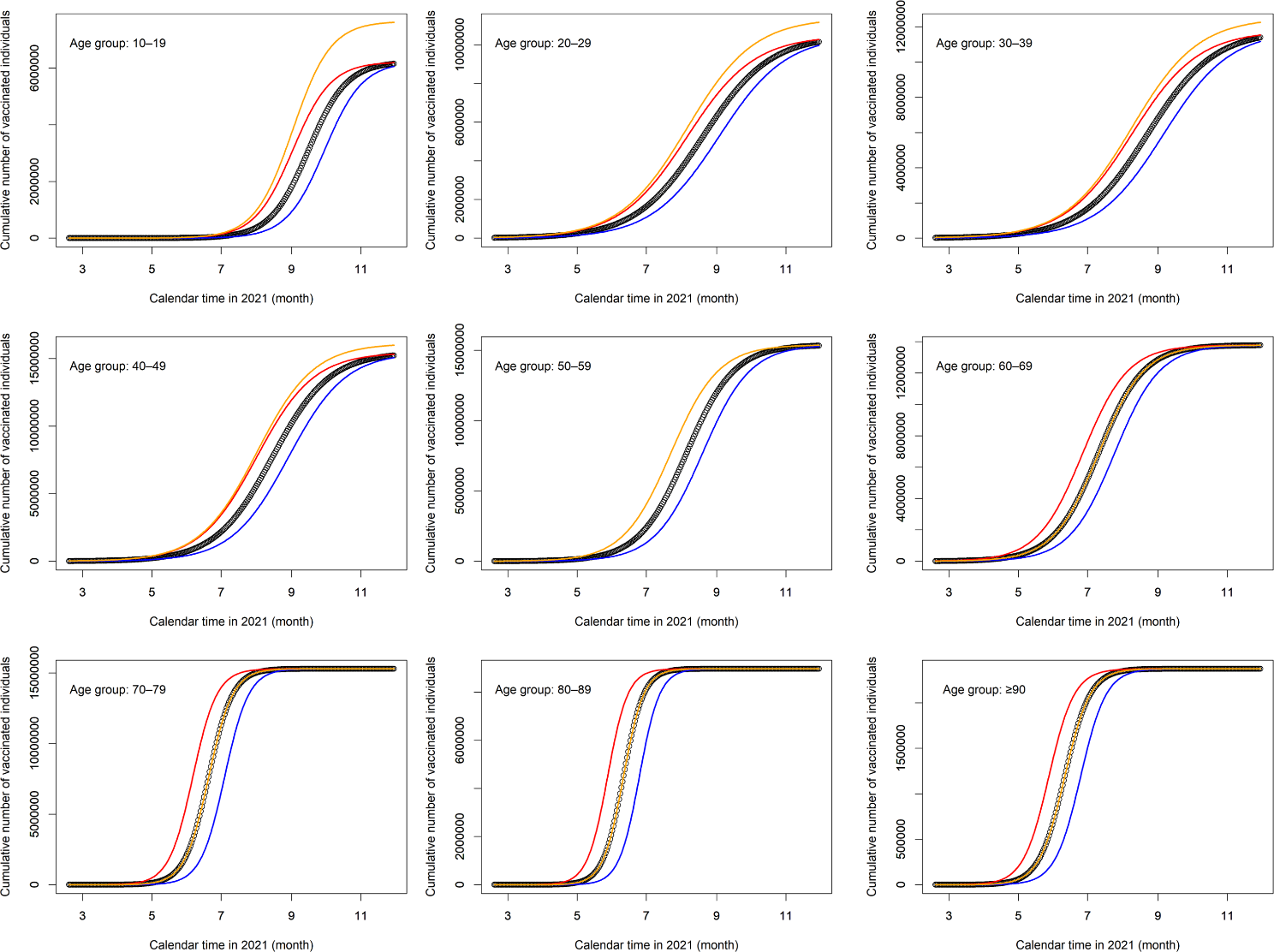
**

**Supplementary Figure S7. Counterfactual scenarios of number of vaccinated people in the primary series of the vaccination program by age group.**

Black circles represent the numbers of vaccinated individuals replicated in the model. Red, blue, and orange lines indicate the Early vaccination scenario, the Late vaccination scenario, and the Elevated vaccination scenario, respectively.

**
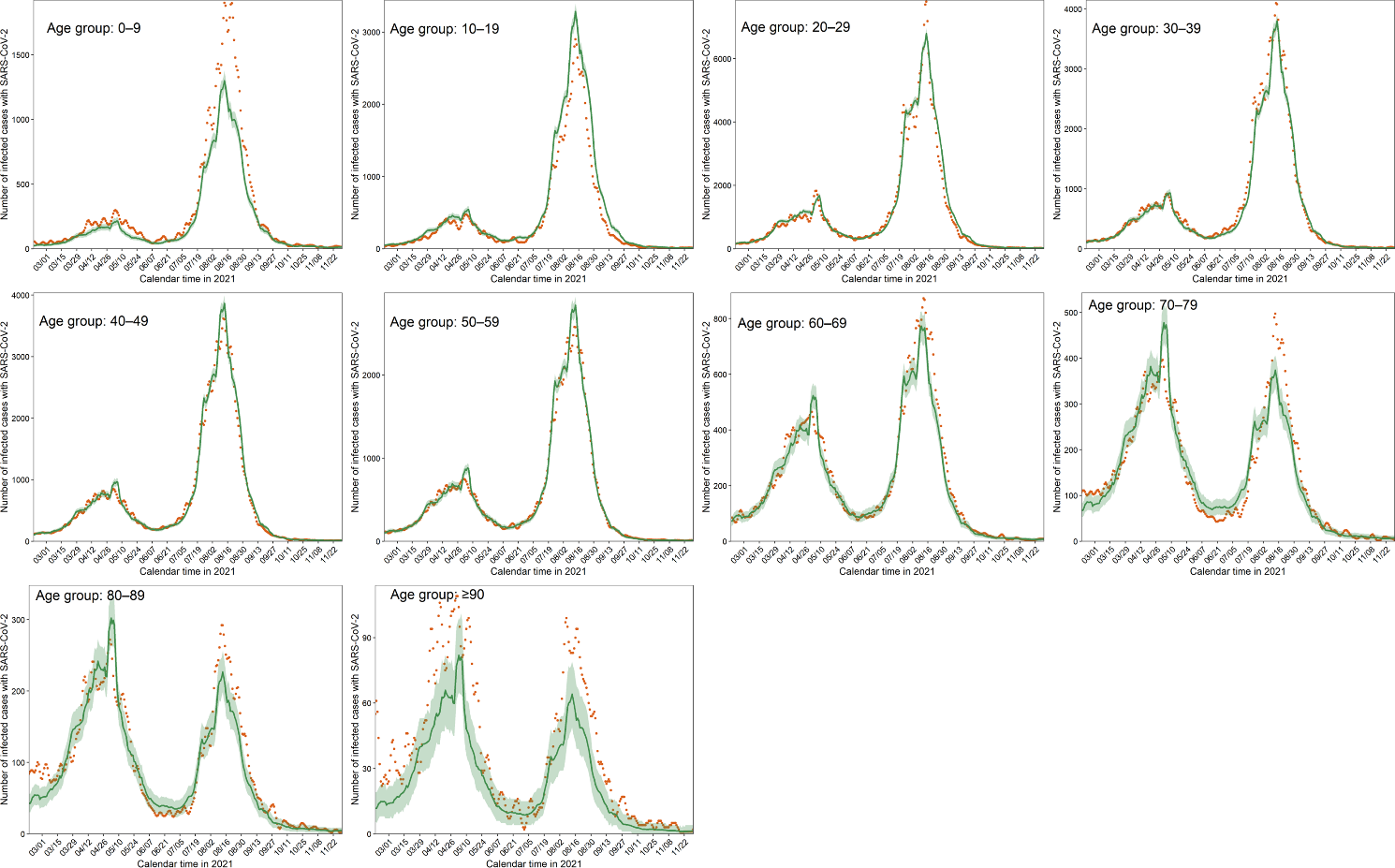
**

**Supplementary Figure S8.** **Comparison between predicted and observed infections with SARS-CoV-2 by age group.**

Orange dots represent the observed daily incidence of SARS-CoV-2 infection during the primary series of the vaccination program from 17 February to 30 November 2021. Green line denotes the predicted daily incidence in the transmission model, with 95% confidence intervals highlighted as light green areas. The observed incidence of COVID-19 by age group is the same as the number of confirmed cases, assuming no ascertainment bias exists.


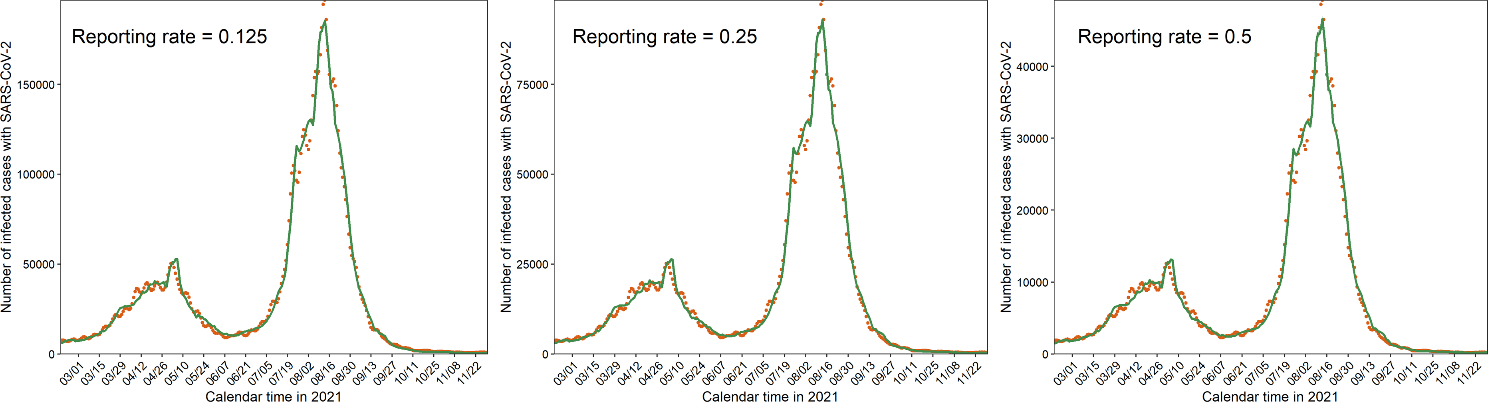


**Supplementary Figure S9.** **Comparison between predicted and observed infections with SARS-CoV-2 by reporting coverage.**

Orange dots represent the daily incidence of SARS-CoV-2 infection during the primary series of the vaccination program from 17 February to 30 November 2021. Green line denotes the predicted daily incidence in the transmission model, with 95% confidence intervals highlighted as light green areas. Supplementary Figure shows comparisons with reporting coverages of 0.125, 0.25, and 0.50.


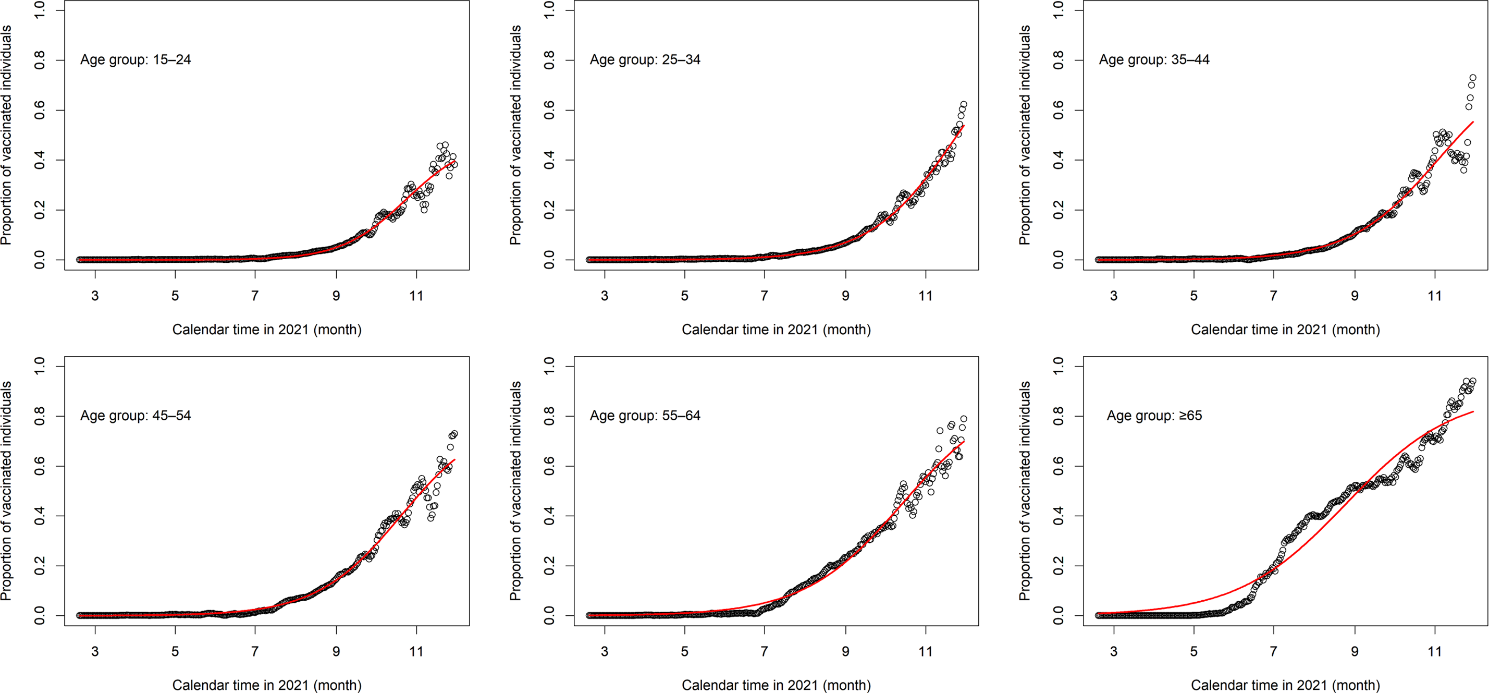


**Supplementary Figure S10.** **Comparison between predicted and observed proportion of vaccinated people among confirmed COVID-19 cases by age group.**

Black circles represent the observed daily proportion of vaccinated people among all confirmed COVID-19 cases. Red lines describe the daily proportion predicted from the logistic function.

**
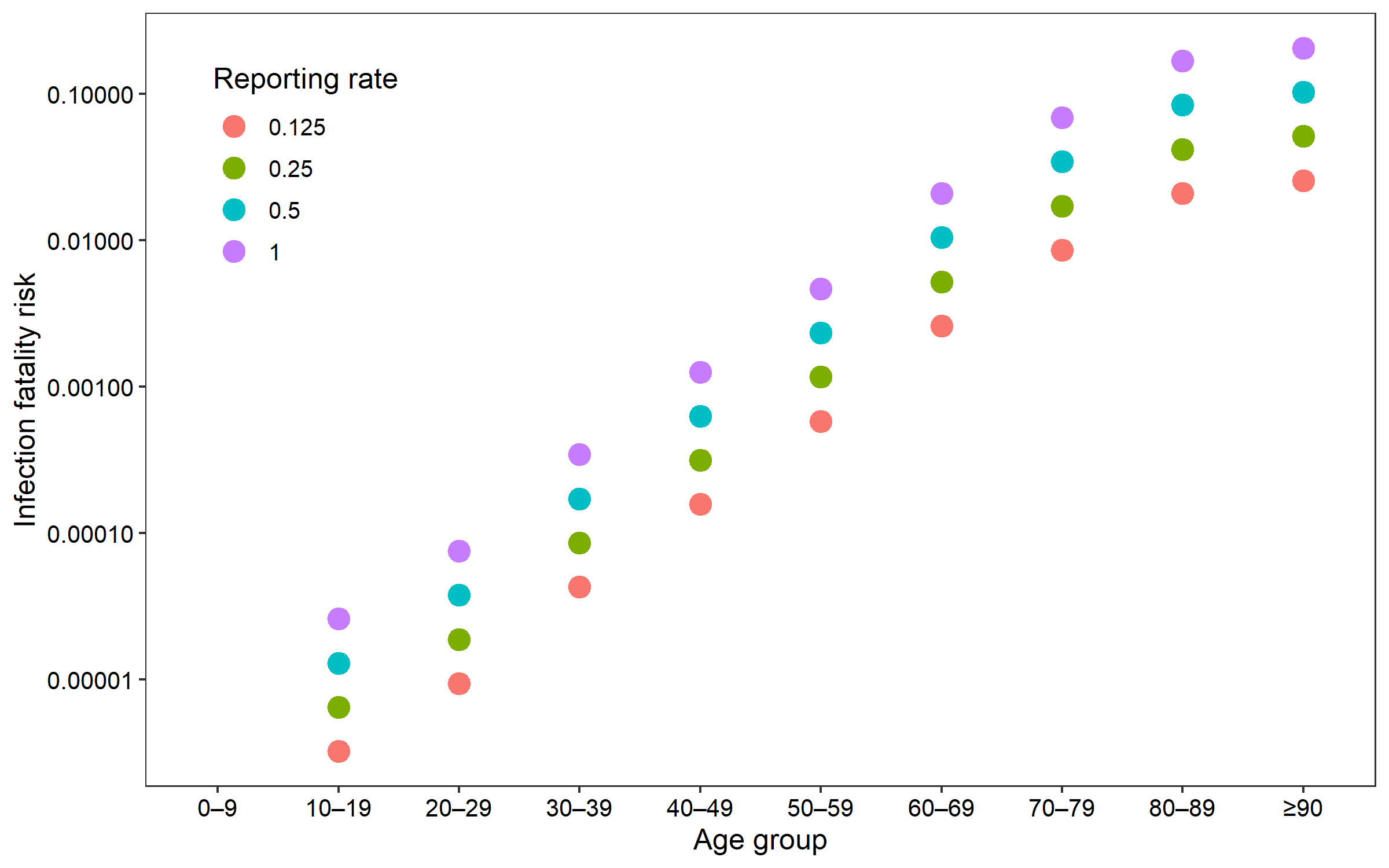
**

**Supplementary Figure S11. Infection fatality risk by reporting coverage and age group.**

Infection fatality risk by age group was estimated according to the cumulative number of estimated infections in each reporting coverage. The vertical axis represents the infection fatality risk with a logarithmic scale to the base 10.

**Supplementary Table S1. Estimated parameters by reporting coverage.**

| **Parameter** | **Description** | **Reporting coverage** | **Estimated value** |
| --- | --- | --- | --- |
| $p$ | Overall scaling parameter | 1 | 2.749 (2.718 – 2.782)^a^ |
|  |  | 0.5 | 2.722 (2.699 – 2.745) |
|  |  | 0.25 | 2.660 (2.644 – 2.677) |
|  |  | 0.125 | 2.509 (2.497 – 2.521) |
| $\omega_{h}$ | Coefficient expressing mobility in the house setting  (relative to the community setting) | 1 | −0.490 (−0.496 – −0.485) |
|  |  | 0.5 | −0.483 (−0.487 – −0.479) |
|  |  | 0.25 | −0.467 (−0.470 – −0.464) |
|  |  | 0.125 | −0.427 (−0.430 – −0.425) |
| $\omega_{w}$ | Coefficient expressing mobility in the work setting  (relative to the community setting) | 1 | −0.083 (−0.090 – −0.077) |
|  |  | 0.5 | −0.088 (−0.092 – −0.083) |
|  |  | 0.25 | −0.096 (−0.099 – −0.093) |
|  |  | 0.125 | −0.118 (−0.121 – −0.116) |
| $r$ | Transmissibility scaling in the Delta variant | 1 | 0.994 (0.990 – 0.999) |
|  |  | 0.5 | 1.005 (1.002 – 1.008) |
|  |  | 0.25 | 1.029 (1.026 – 1.031) |
|  |  | 0.125 | 1.080 (1.079 – 1.082) |
| $e$ | Effect of consecutive holidays  (more than two days and Obon season^b^) | 1 | 1.018 (1.012 – 1.025) |
|  |  | 0.5 | 1.017 (1.013 – 1.021) |
|  |  | 0.25 | 1.015 (1.011 – 1.018) |
|  |  | 0.125 | 1.009 (1.007 – 1.011) |

^a^ The 95% confidence intervals estimated using the bootstrap method are in parentheses.

^b^ Traditional holidays during summer in Japan.
